# The clinical course of COVID-19 in hematopoietic stem cell transplantation (HSCT) recipients

**DOI:** 10.3906/sag-2103-72

**Published:** 2021-08-30

**Authors:** Ayşe KARATAŞ, Ümit Yavuz MALKAN, Mustafa VELET, Haluk DEMİROĞLU, Yahya BÜYÜKAŞIK, Gülçin TELLİ DİZMAN, Ahmet Çağkan İNKAYA, Batuhan ERDOĞDU, Olgu Erkin ÇINAR, Elifcan ALADAĞ, Salih AKSU, İbrahim Celalettin HAZNEDAROĞLU, Osman ÖZCEBE, Nilgün SAYINALP, Hakan GÖKER

**Affiliations:** 1 Division of Hematology, Department of Internal Medicine, Faculty of Medicine, Hacettepe University, Ankara Turkey; 2 Department of Infectious Diseases, Faculty of Medicine, Hacettepe University, Ankara Turkey; 3 Division of Hematology, Department of Internal Medicine, Faculty of Medicine, University of Health Sciences, Dışkapı Yıldırım Beyazıt Training and Research Hospital, Ankara Turkey; 4 Division of Hematology, Department of Internal Medicine, Faculty of Medicine, Gülhane Training and Research Hospital,University of Health Sciences, Ankara Turkey

**Keywords:** Hematopoietic stem cell transplantation, bone marrow transplant, COVID-19

## Abstract

**Background/aim:**

The disease caused by SARS-CoV-2 was named as COVID-19. There is as yet insufficient information about the effects of HSCT on the clinical course of COVID-19. In the present study, we aimed to investigate the clinical course of COVID-19 in patients who had undergone HSCT.

**Materials and methods:**

We analyzed baseline characteristics, clinical course and findings of COVID-19, hospitalization and death rates, overall survival, and case fatality rates of HSCT recipients diagnosed with COVID-19 retrospectively.

**Results:**

57.6% of the patients underwent AHSCT, and 42.4% underwent allo-HSCT. 60.6%, 27.3%, and 12.1% of the patients had mild, moderate, and severe COVID-19 or critical illness, respectively. Overall, 45.5% were hospitalized, 12.1% required intensive care, and 9.1% necessitated invasive mechanical ventilation. The total CFR was 9.1% in HSCT recipients, 22.2% in patients with active hematologic malignancy, and 4.2% in patients without active hematologic malignancy.

**Conclusion:**

It can be concluded that mortality of HSCT recipients is lower in patients whose primary disease is in remission compared to ones that are not in remission. Further studies with larger group patients are needed in order to delineate the effects of COVID-19 on HSCT patients.

## 1. Introduction

The disease caused by severe acute respiratory syndrome coronavirus 2 (SARS-CoV-2) was first discovered in Wuhan, China and named as coronavirus disease 2019 (COVID-19) then spread all over the world, and declared as a pandemic by the World Health Organization [1].

Clinical course of the disease varies from asymptomatic disease to significant morbidity and mortality due to respiratory failure. Advanced age, male sex, hypertension, diabetes mellitus, cardiovascular diseases, and cancer have been shown as risk factors worsening outcomes. There are studies that show higher mortality rates in hematologic cancer patients [2,3]. The characteristics of the disease course in immunosuppressive patients are not exactly known yet [4]. Hematopoietic stem cell transplantation (HSCT) recipients are considered to be at risk due to severe immunosuppressive therapy and immune system dysregulation after HSCT [5]. There is as yet insufficient information about the effects of COVID-19 on the clinical course of HSCT patients. In the present study, we aimed to investigate the effects of COVID-19 in patients who had undergone HSCT.

## 2. Materials and methods

According to the hospital medical records, 33 patients followed in the stem cell transplantation unit of Hacettepe University Hematology Department were diagnosed with COVID-19 between 11th March and 30th November 2020. The data of these patients was analyzed retrospectively. COVID-19 was diagnosed with positive result of reverse-transcriptase polymerase chain reaction (RT-PCR) test from upper respiratory samples or bronchoalveolar lavage fluid. The disease with only mild symptoms (cough, fever, anosmia, fatigue) was evaluated as mild disease. The disease with clinical or radiological findings of pneumonia, but not requiring supportive oxygen therapy was evaluated as moderate disease. Patients with pneumonia and oxygen saturation below 93% or respiratory rate above 30/min were considered to have severe COVID-19. The condition of the patients with respiratory failure, shock or multi-organ failure was evaluated as critical illness [6]. Some high-risk patients with mild COVID-19 were hospitalized for observation purposes only. 

The approvals were obtained from the Turkish Ministry of Health and the local ethics committee with decision number 2020/20-54.

IBM SPSS Statistics for Windows v: 25.0 (IBM Corp., Armonk, NY, USA) was used for statistical analyses. Categorical data was analyzed using Chi-square or Fisher’s exact test. The distribution of continuous data was examined. Mean ± standard deviation and median (minimum-maximum) values were given for normally distributed continuous and nonnormally distributed variables, respectively. At the time of last follow-up, patients who were alive were censored for overall survival analysis. The Kaplan–Meier method was used for overall survival analysis.

## 3. Results

Thirty-three patients were included in the study. Median age was 57 (27–71) years. Nineteen (57.6%) of the patients underwent autologous hematopoietic stem cell transplantation (AHSCT), and 14 (42.4%) underwent allogeneic hematopoietic stem cell transplantation (Allo-HSCT). Nine (27.3%) had uncontrolled primary disease, and 4 (12.1%) were on calcineurin inhibitors at the time of COVID-19 diagnosis. Twelve (85.7%) of allo-HSCT recipients were transplanted from 10/10 human leukocyte antigen (HLA) matched related donors, two of them (14.3%) from haploidentical donors. The patients baseline characteristics are outlined in Table 1.

**Table 1:** Baseline characteristics of the patients*.

Median Age (min-max)	57 (27-71) years
Gender, n (%)	
Male	21 (63.6%)
Female	12 (36.4%)
Time from HSCT to COVID-19 Median (min-max)	868 (31-2368) days
Primary Disease (Requiring HSCT), n (%)	
MM	9 (27.3%)
HL	1 (3.0%)
NHL	8 (24.2%)
AML	5 (15.2%)
ALL	3 (9.1%)
PID	2 (6.1%)
MF	1 (3.0%)
MDS	3 (9.1%)
ITP	1 (3.0%)
HSCT, n (%)	
AHSCT	19 (57.6%)
Allo-HSCT	14 (42.4%)
Uncontrolled Primary Disease, n (%)	9 (27.3%)
Use of Immunosuppressive Drugs, n (%) (Calcineurin inhibitors)	4 (12.1%)
Donor (n=14)	
Full matched donor, n (%)	12 (83.3%)
Haploidentical, n (%)	2 (16.7%)
GVHD, n (%)	6 (18.2%)
Chronic Diseases, n (%)	
CAD	4 (12.1 %)
CKD	7 (21.2%)
HT	8 (24.2 %)
COPD	1 (3.0 %)
DM	3 (9.1 %)
Non-hematologic malignancy	2 (6.1 %)

*MM: multiple myeloma, HL: hodgkin-lymphoma: NHL: nonhodgkin lymphoma, AML: acute myeloid leukemia, ALL: acute lymphoblastic leukemia, PID: primary immunodeficiency, MF: mycosis fungoides, MDS: myelodysplastic syndrome, ITP: immune thrombocytopenia, GVHD: graft versus host disease, CAD: coronary artery disease, CKD: chronic kidney disease, HT: hypertension, COPD: chronic obstructive pulmonary disease, DM: diabetes mellitus.

Among the patients, 23 (69.7%) of them developed fever in the course of COVID-19. Twenty (60.6%), 9 (27.3%), and 4 (12.1%) of the patients had mild, moderate and severe COVID-19 or critical illness, respectively. Fifteen (45.5%) were hospitalized, 4 (12.1%) required intensive care, and 3 patients (9.1%) needed invasive mechanical ventilation. A patient, who refused hospitalization died at home. Favipiravir, which is a ribonucleic acid polymerase inhibitor, was the most common antiviral medication used by the patients. Detailed COVID-19 outcomes of the patients are given in Table 2.

**Table 2:** The clinical findings in HSCT patients.

Symptoms	
Fever	23 (69.7%)
Cough	14 (42.4%)
Myalgia	18 (54.6%)
Headache	19 (57.6%)
Anosmia	14 (42.4%)
Diarrhea	5 (15.2%)
Sore throat	4 (12.1%)
Shortness of Breath	10 (30.3%)
COVID-19 Severity	
Mild	20 (60.6 %)
Moderate	9 (27.3 %)
Severe or Critical Disease	4 (12.1 %)
Hospitalization	
n (%)	15 (45.5%)
Days, median (min-max)	12 (1-57) days
Treatment for COVID-19 (n, %)	
Hydroxychloroquine + Favipiravir	5 (15.6%)
Favipiravir	24 (75%)
Azithromycin + Hydroxychloroquine	3 (9.4%)
Intensive Care Unit Admission	
n (%)	4 (12.1%)
Days (mean ± SD)	15.2 ± 12.7
Mechanical Ventilation, n (%)	3 (9.1%)
Case Fatality Rate	3/33 (9.1%)
Duration to Death from COVID-19 (days) (mean ± SD)	14.6 ± 6.8

A total of 6 patients had concomitant graft versus host disease (GVHD) (acute gastrointestinal and cutaneous: two patients, chronic cutaneous and gastrointestinal: two patients, chronic cutaneous: two patients) at the time of COVID-19 diagnosis. The course of the GVHD did not get worse in these patients.

Among the hospitalized patients, 13 (86.7%) of them were male, and the remaining 2 (13.3%) were female. The difference was statistically significant (p < 0.05). Hospital admission rates according to sex, HSCT type, remission status, and presence of GVHD are given in Table 3.

**Table 3:** Hospitalization rates according to parameters.

Patients	Hospitalized	Non-Hospitalized	P-value
Gender			0.01
Female (n = 12)	2 (13.3%)	10 (55.6 %)	
Male (n = 21)	13 (86.7%)	8 (44.4 %)	
HSCT			0.65
AHSCT	8 (53.3%)	11 (61.1 %)	
Allo-HSCT	7 (46.7%)	7 (38.9 %)	
Remission Status of Primary Disease			0.47
Yes	7 (46.7%)	2 (11.1%)	
No	8 (53.3%)	16 (88.9%)	
GVHD (in Allo-HSCT patients, n = 14)			0.59
Yes (n = 6)	2 (28.6 %)	4 (57.1 %)	
No (n = 8)	5 (71.4 %)	3 (42.9 %)	

Median follow-up time was 28 days (2–262 days). During this time, 3 of 33 patients (9.1%) died. The case fatality rate was 9.1% in all HSCT recipients, 22.2% in patients with active hematologic malignancy, 4.2% in patients without active hematologic malignancy, and 25.0% in who was on immunosuppressive drugs. The mortality rates according to sex, HSCT type, remission status, presence of GVHD, and use of immunosuppressive drugs are given in Table 4.

**Table 4:** The mortality rates according to parameters.

Patients(n = 33)	Death(n = 3)	Survivor(n = 30)	P- value
Gender			0.2
Male	3 (100.0%)	18 (60.0%)	
Female	0 (0.0%)	12 (40.0%)	
HSCT			0.56
AHSCT	1 (33.3%)	18 (60.0%)	
Allo-HSCT	2 (66.7%)	12 (40.0%)	
Remission of Primary Disease			0.174
Yes (n = 9)	2 (66.7%)	7 (23.3%)	
No (n = 24)	1 (33.3%)	23 (76.7%)	
Use of Immunosuppressive Drug			0.33
Yes (n = 4)	1 (33.3%)	3 (10.0%)	
No (n = 29)	2 (66.7%)	27 (90.0%)	
GVHD (n=14)			1.0
Yes (n = 6)	1 (50.0%)	5 (41.7%)	
No (n = 8)	1 (50.0%)	7 (58.3%)	

The mortality and hospitalization rates were calculated separately for the treatments. The detailed data on COVID-19 treatments received by the patients are given in Table 5. The treatment records of a patient who refused hospitalization and treated at home were not available. In univariate survival analysis, there was not statistically significant difference between the AHSCT and allo-HSCT recipients (p = 0.39). Kaplan–Meier survival curve of AHSCT and allo-HSCT recipients is shown in Figure.

**Table 5:** Death and hospitalization rates according to COVID-19 treatment*.

	Hydroxychloroquine + Favipiravirn = 5	Azithromycin + Hydroxychloroquinen = 3	Favipiravirn = 24
Hospitalization	4 (80.0%)	1 (33.3%)	13 (54.2%)
Non-Hospitalized	1 (20.0%)	2 (66.7%)	11 (45.8%)
Dead	0 (0.0%)	0 (0.0%)	3 (12.5%)
Survivor	5 (100.0%)	3 (100.0%)	21 (87.5%)

*For this table n = 32, since the treatment records of a patient were not available.

**Figure:**
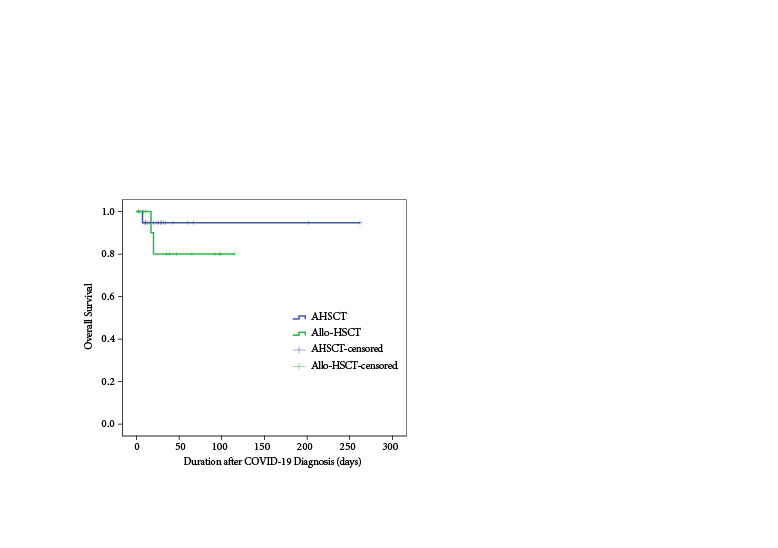
Overall survival after COVID-19 diagnosis.

## 4. Discussion

Information on the course of COVID-19 in HSCT recipients remained scarce, and mostly derived from case series involving limited number of patients. Survival rate of the HSCT recipients is a matter of debate. Sultan et al. and Haroon et al. did not report any mortality among 7 and 11 HSCT recipients, respectively [3,5]. However, Varma et al. published the outcomes of HSCT recipients with COVID-19 where 32% of the patients were treated in the intensive care unit and 21% died [7]. In another case series, 2 of 7 HSCT recipients died due to COVID-19 [8]. In another study conducted in Spain, mortality was 20% in allo-HSCT recipients, and 24% in AHSCT recipients [9]. 

In another study, the CFR was 15.6% in HSCT recipients. The CFR of the HSCT recipients who were receiving immunosuppressive treatment was reported as 33% in that study [10]. The patients on immunosuppressive treatment can be considered at high risk for COVID-19 as well as other viral infections. In a cohort study examining the results of 318 HSCT recipients, overall survival 30 days after the COVID-19 diagnosis was 68% (95% CI 58–77) in allo-HSCT recipients and 67% (95% CI 55–78) in AHSCT recipients [11]. In the present study, case fatality rate (CFR) is 9.1% (3/33) in all HSCT recipients, 22.2% (2/9) in patients with active hematologic malignancy, and 4.2% (1/24) in patients without active hematologic malignancy. All of the patients received favipiravir as soon as the diagnosis was made which could lead to favorable outcome in the present study [12,13]. This study reflects our experience though with a limited number of HSCT recipients, given insight in the outcome of HSCT patients with COVID-19 infection. CFR was 9.1% in all patients, and 4.2% in patients with primary disease in remission. Based on the data above presented, it can be concluded that mortality of HSCT recipients is lower in patients whose primary disease is in remission compared to ones that are not in remission. Further studies with large group patients are needed in order to delineate the effects of COVID-19 in HSCT patients. 

## Informed consent

As the standard action of Hacettepe University Faculty of Medicine hospitals, it was confirmed from the patient records that all patients included in the study gave informed consent for the standard diagnosis and treatment procedures at the time of admission to the hospital. The approvals were obtained from the Turkish Ministry of Health and the local ethics committee with decision number 2020/20-54.
